# Cadmium Caused Different Toxicity to Photosystem I and Photosystem II of Freshwater Unicellular Algae *Chlorella* *pyrenoidosa* (Chlorophyta)

**DOI:** 10.3390/toxics10070352

**Published:** 2022-06-28

**Authors:** Shuzhi Wang, Rehemanjiang Wufuer, Jia Duo, Wenfeng Li, Xiangliang Pan

**Affiliations:** 1 National Engineering Technology Research Center for Desert-Oasis Ecological Construction, Xinjiang Institute of Ecology and Geography, Chinese Academy of Sciences, 818 South Beijing Road, Urumqi 830011, China; wangshuzhi@ms.xjb.ac.cn (S.W.); reheman319@ms.xjb.ac.cn (R.W.); duojia2017@ms.xjb.ac.cn (J.D.); 2 Xinjiang Key Laboratory of Environmental Pollution and Bioremediation, Xinjiang Institute of Ecology and Geography, Chinese Academy of Sciences, Urumqi 830011, China; 3 Key Laboratory of Microbial Technology for Industrial Pollution Control of Zhejiang Province, College of Environment, Zhejiang University of Technology, Hangzhou 310014, China

**Keywords:** *Chlorella* *pyrenoidosa*, cadmium, photosystem I, photosystem II, electron transport rate, cyclic electron flow

## Abstract

Heavy metals such as Cd pose environmental problems and threats to a variety of organisms. The effects of cadmium (Cd) on the growth and activities of photosystem I (PSI) and photosystem II (PSII) of *Chlorella* *pyrenoidosa* were studied. The growth rate of cells treated with 25 and 100 µM of Cd for longer than 48 h were significantly lower than the control, accompanying with the inhibition of photosynthesis. The result of quantum yields and electron transport rates (ETRs) in PSI and PSII showed that Cd had a more serious inhibition on PSII than on PSI. Cd decreased the efficiency of PSII to use the energy under high light with increasing Cd concentration. In contrast, the quantum yield of PSI did not show a significant difference among different Cd treatments. The activation of cyclic electron flow (CEF) and the inhibition of linear electron flow (LEF) due to Cd treatment were observed. The photochemical quantum yield of PSI and the tolerance of ETR of PSI to Cd treatments were due to the activation of CEF around PSI. The activation of CEF also played an important role in induction of non-photochemical quenching (NPQ). The binding features of Cd ions and photosystem particles showed that Cd was easier to combine with PSII than PSI, which may explain the different toxicity of Cd on PSII and PSI.

## 1. Introduction

Heavy metal pollution is one of the most important environmental problems and poses a serious threat to the ecosystem and human health [1,2]. Metal compounds are also one of the most persistent pollutants in aquatic environments [3]. Among heavy metals, cadmium (Cd) is a highly toxic element for plants and humans [4]. Cd exposure causes the inhibition of growth and decrease of the yield of crops [5], and Cd decreases photosynthetic activity by decreasing the pigment content and stomatal conductance, with the destruction of chlorophyll structure and the direct toxicity of reactive oxygen species (ROS) [4,6].

Cd is one of the major metal pollutants because of its wide distribution in aquatic ecosystems and its high toxicity [7,8]. In recent years, phytoplankton species, including cyanobacteria and green alga, have been widely used as experimental materials in ecological risk assessments to evaluate the impacts of metal, herbicide, and other contamination in aquatic systems [9,10,11], and the effects of heavy metals on alga have been extensively studied [12,13]. Many studies showed that heavy metals had adverse effects on photosystem II (PSII) activities, such as the capture and transfer of energy, quantum yield of photochemistry, and the inhibition of the gene that codes for the integral membrane protein D1 of PSII [14,15,16,17]. PSII is a large pigment-protein complex in the thylakoid membrane, whose main function is the light-induced oxidation of water [18]. On light absorption, the reaction center chlorophyll of PSII at the singlet excited state derives electrons from water. The electrons are transported to photosystem I (PSI) through the electron transport chain, then to NADP and the Calvin cycle [19,20]. Cyclic electron flow (CEF) around PSI operates on the reducing side of PSI via electron transfer between the soluble electron acceptor of PSI and the cytochrome (Cyt) b*_6_*f complex or plastoquinone pool [21]. CEF around PSI involves two pathways: either proton gradient regulation-dependent or chloroplastic NAD(P)H dehydrogenase-dependent cyclic flow [19]. CEF can feed electrons to the plastoquinone pool or Cyt b*_6_*f complex, which in turn reduces the donor side of PSI [22].

The studies investigated the effects of heavy metals on photosystems and suggested that PSI is less sensitive to heavy metals than PSII [23,24]. A few reports have shown the effects of cadmium (Cd) on the activity of photosystem I (PSI), some of which have been controversial. Zhou et al. (2006) [8] noticed an increase of PSI activity when *Microcystis aeruginosa* was treated with elevated levels of Cd. However, another study reported that Cd inhibited PSI activity in *Microcystis* sp. [25]. Results derived from *Synechocystis* demonstrated no Cd effect on parameter for representing the quantity of efficient PSI complexes in cyanobacteria in vivo, and some changes in PSI appeared to be positive [26]. However, some recent findings have suggested that the inhibition of PSII by Cd was more pronounced than that of PSI [27,28]. Thus, the effects of Cd on the activities of PSI and PSII and the differences of the toxicity of Cd on the two photosystems need to be further studied.

To our knowledge, the effects of heavy metals on PSII and PSI are separately studied in most studies, and the toxicity of heavy metals to PSI and PSII photosynthetic activities has been rarely analyzed simultaneously, mainly due to the difficulty in measuring PSI and PSII activities at the same time. An improved method, using saturating light pulses for the determination of PSI quantum yield via P700^+^-absorbance changes at 830 nm, was developed afterwards [29]. A Dual-PAM-100 system, which could measure chlorophyll fluorescence and P700^+^ absorbance changes simultaneously, was introduced and used in some studies [30,31,32], which will be an accurate, rapid, and efficient tool for toxicity bioassays. CEF around PSI was suggested to be important for photoprotection and photosynthesis [32,33]. The physiological role of CEF has been studied under some conditions [20,31,34], and the response of CEF to heavy metals such as Cd and Cr were preliminarily studied [35,36]. However, the effects of heavy metals on CEF were still not well studied.

Microalgae such as *Chlorella* sp. have shown their priority in the field of environmental remediation, and their application and the mechanism of removing heavy metals from water were well discussed [37,38]. The aim of this work was to detect the effects of Cd on the growth and activities of PSI and PSII and CEF of the green alga *Chlorella pyrenoidosa*. The differences of the toxicity of Cd on quantum yield and electron transport rate of the two photosystems were compared. The results showed that Cd mainly caused serious inhibitory effects on PSII, while the activity of PSI showed resistance to Cd exposure in the present study. The reason for the difference was further explained by studying the binding ability between Cd and isolated photosystems.

## 2. Materials and Methods

### 2.1. Culture of Chlorella pyrenoidosa

*Chlorella pyrenoidosa* (FACHB-9) was purchased from the Freshwater Algae Culture Collection of Institute of Hydrobiology, Chinese Academy of Sciences (Wuhan, China). The algal cells were cultured in BG-11 medium [39] at 25 °C under a fluorescent white light (30 μmol m^−2^ s^−1^) with a 12:12 h light:dark cycle. The growth of cultures was monitored every day by measuring the cell optical density at 680 nm (OD_680_) with a UV-vis spectrophotometer (UV2800, Unico, Shanghai, China). The cells in the exponential growth phase were harvested for Cd treatment experiments.

### 2.2. Cd Treatments

Exponentially growing cells were harvested and cultured in 50-mL flasks with 25 mL of BG-11 medium containing various concentrations of Cd (0, 1, 25, and 100 μM). Cadmium was applied in the form of analytical-grade CdCl_2_. The Cd containing solutions were prepared just before the experiment by dissolving CdCl_2_ in sterilized BG-11 medium and then diluted to the desired concentrations. Cd treatments were carried out with 16 cultures simultaneously: 4 cultures in absence of Cd, 4 cultures at 1 µM Cd, 4 cultures at 25 µM Cd, and 4 cultures at 100 µM Cd. The samples without Cd were used as the control. Algal cells were cultured at 25 °C under a fluorescent white light (30 μmol m^−2^ s^−1^) with a 12:12 h light: dark cycle.

### 2.3. Measurement of Cell Growth

After the cells were exposed to various concentrations of Cd for 0, 6, 12, 24, 48, 72, and 96 h, the optical density at 680 nm (OD_680_) of the samples were measured with a UV-vis spectrophotometer (UV2800, Unico, Shanghai, China) to monitor the cell growth.

### 2.4. Measurement of PSI and PSII Activities

#### 2.4.1. Application of the Dual-PAM-100 System

After the onset of Cd treatments for 72 h, the OD_680_ of the cultures with different treatments was adjusted to around 0.8 with BG-11 medium and used to test the activities of PSI and PSII by a Dual-PAM-100 system (Heinz Walz GmbH, Effeltrich, Germany) [31,32]. PSII and PSI activities were quantified by chlorophyll fluorescence and P700^+^ absorbance changes, respectively [35]. The cells used for the measurements were injected into the DUAL-K25 quartz glass cuvette, which was supplied with the Dual-PAM-100 system and developed to reduce baseline drifts caused by particle settling in suspensions of isolated chloroplasts, unicellular algae, and cyanobacteria. The quartz glass cuvette with algae solution inside was then sandwiched between the emitter head and detector head of the system. The measurements were performed using the automated induction program provided by the Dual-PAM software [40] with a slight modification. All the samples were dark-adapted for 5 min before each test. The minimal fluorescence after dark-adaptation, Fo, was detected by a measuring light at low intensity. A saturating pulse with a duration of 300 ms and a light intensity of 10,000 μmol m^−2^ s^−1^ were then applied to detect the maximum fluorescence after dark-adaptation, Fm. The maximal change in P700^+^ signal, Pm, was determined through the application of a saturation pulse after far-red pre-illumination for 10 s.

#### 2.4.2. Measurement of the Rapid Light Response Curves

After the determination of Fo, Fm, and Pm, the activities of PSI and PSII were tested during the light response reaction, during which the rapid light response curves (RLCs) of quantum yields and electron transport rates were performed in the rapid light curve mode (RLC mode) with the routine of the Dual-PAM software. After the RLC mode was turned on, the actinic light was applied at each photosynthetic active radiation (PAR, which was measured as the photosynthetic photon flux density) for 30 s with increasing intensity (0, 30, 46, 77, 119, 150, 240, 363, 555, 849, and 1311 μmol m^−2^ s^−1^). A saturating pulse was applied after each period of actinic light to determine the maximum fluorescence signal (Fm′) and maximum P700^+^ signal (Pm′) under the actinic light. RLC with saturation pulse analysis was based on the previously determined Fo, Fm, and Pm.

#### 2.4.3. Quantum Yields of Photosystems and CEF

The quantum yields of PSI and PSII were detected by saturating pulses after each PAR during the light response reaction and calculated automatically by the Dual-PAM software. The quantum yields of energy conversion in PSII were calculated according to the method as described previously, which can be transformed into the following simpler equations [32]: Y(II) = (Fm′ − F)/Fm′, Y(NPQ) = F/Fm′ − F/Fm, and Y(NO) = F/Fm, where F was the steady state fluorescence, Y(II) was the effective photochemical quantum yield of PSII, Y(NPQ) was the quantum yield of light-induced non-photochemical fluorescence quenching (NPQ), and Y(NO) was the quantum yield of non-light-induced non-photochemical fluorescence quenching. These quantum yields are complementary, i.e., they add up to one: Y(II) + Y(NPQ) + Y(NO) = 1 [40].

The P700^+^ signal (P) was recorded just before a saturation pulse then again briefly after the onset of a Saturation Pulse (Pm′), when the maximum P700 oxidation under the effective experimental conditions was observed, and finally at the end of the 1 s dark interval following each saturation pulse (Po determination). The signals P and Pm′ were detected referenced against Po. Pm, Pm′, and Po were used in the calculation of the quantum yields of energy conversion in PSI: Y(I) = (Pm′ − P)/Pm, Y(ND) = (P − Po)/Pm, Y(NA) = (Pm − Pm′)/Pm, where Y(I) was an effective photochemical quantum yield, Y(ND) was the quantum yield of non-photochemical energy dissipation in reaction centers due to donor side limitation, and Y(NA) was the quantum yield of non-photochemical energy dissipation of the reaction centers due to acceptor side limitation. These three quantum yields are complementary: Y(I) + Y(ND)+ Y(NA) = 1 [40].

The quantum yield of CEF was calculated from Y(I) and Y(II): Y(CEF) = Y(I) − Y(II) [31].

The ratios of Y(CEF)/Y(I), Y(II)/Y(I), and Y(CEF)/Y(II) were calculated to show the change of the distribution of quantum yields between the two photosystems and the change of the ratio of quantum yield of CEF to that of LEF [31].

#### 2.4.4. Analysis of RLCs of Electron Transport Rates in PSI and PSII

Electron transport rates in PSI and PSII, i.e., ETR(I) and ETR(II), were defined and calculated using the Dual-PAM software. Descriptive parameters of ETR(I) and ETR(II) during the light response reaction were derived from the RLCs, which were automatically calculated by the Dual-PAM software according to the exponential function referring to Platt et al. (1980) [41]. The parameters calculated by the Dual-PAM software were as follows: α, the initial slope of RLC of ETR(I) or ETR(II), which reflected the photochemical efficiency [42]; ETRmax, the maximal electron transport rates in PSI or PSII; Ik, the index of the light adaptation of PSI or PSII (i.e., the irradiance at onset of saturation), was calculated as ETRmax/α [41,43].

### 2.5. Isolation of Photosystems and the Binding with Cd Ions

The culture of *C. pyrenoidosa* was centrifugated at 5000× *g* for 10 min to harvest the cells for the preparation of thylakoid membranes and the isolation of PS particles. Thylakoid membranes were isolated using the following protocol from Fisher et al. (1997) [44]. PSI and PSII were isolated by the methods described previously [45,46].

For the purification of PSI, thylakoid membranes were diluted to 0.8 mg Chl/mL in solubilization buffer: 25 mM Hepes-KOH, 100 mM NaCl, 5 mM MgSO_4_, 10% glycerol (pH 7.5), solubilized by the addition of 1/10 volume of 10% n-Dodecyl β-d-maltoside (DDM; Anatrace Inc., Maumee, OH, USA), and then gently mixed in the dark (30 min, 4 °C). After centrifugation at 46,000× *g* for 25 min at 4 °C, the supernatant was harvested and loaded onto a HisTrap HP Ni2+ column pre-equilibrated with solubilization buffer + 0.03% DDM and washed with the same buffer with 2 mM of imidazole until the UV absorbance reached a baseline. PSI was then eluted by 1 column volume of 25 mM MES-NaOH, 100 mM NaCl, 5 mM MgSO_4_, 10% glycerol, 0.03% DDM, 300 mM imidazole (pH 7.0) buffer.

For the isolation of PSII, thylakoid membranes were resuspended in buffer with 25 mM MES, pH 6.5, 100 mM NaCl, 10% (*v*/*v*) glycerol, 10 mM ascorbic acid, plus 500 μL of protease inhibitor cocktail, Sigma, Welwyn Garden City, UK. PSII was solubilized by using 25 mM of DDM in buffer as above in the previous step and centrifuged at 11,000× *g* for 10 min to remove debris. The supernatant was added to washed nickel resin (Qiagen) in washing buffer: 25 mM MES, pH 6.5, 100 mM NaCl, 10% glycerol, 0.03% (*w*/*v*) DDM plus protease inhibitors, 10 mM ascorbic acid, and 5 mM imidazole. After 30 min, the slurry was poured into a chromatography column and washed with washing buffer until the eluate was visually clear. PSII was eluted with 40 mM MES, pH 6.0, 100 mM NaCl, 10% glycerol, 0.03% DDM, 10 mM ascorbic acid, and 200 mM imidazole and mixed with an equal volume of 20 mM MES, pH 6.3, 15 mM NaCl, 20% (*w*/*v*) PEG 8000, 10 mM ascorbic acid, and 10 mM EDTA. The precipitate was pelleted by centrifugation at 11,000× *g* for 10 min. The pellet was resuspended in 20 mM of MES pH 6.3, 15 mM NaCl, 10 mM ascorbic acid, 10% PEG, 10 mM CaCl2, and centrifuged again at 11,000× *g* for 10 min to decrease the imidazole concentration. The isolated PSII was finally resuspended in the preservation solution as follows.

PSI particles were resuspended in the PSI preservation solution (20 mM Tricine-NaOH, 10 mM NaCl, 10 mM KCl, and 5 mM MgCl_2_; pH = 7.8). PSII particles were resuspended in the PSII preservation solution (0.4 M sucrose, 5 mM MgCl_2_, 10 mM NaCl, 40 mM MES-NaOH; pH = 6.5). Samples were taken from the separated photosystem suspension for chlorophyll concentration determination according to [47]. The isolated PSI and PSII complexes were immediately used in the next experimental step or stored at −20 °C for the next experiments in short term.

The 3D fluorescence of two photosystems was determined using a fluorescence spectrophotometer (F-7000, Hitachi, Tokyo, Japan) at 298 K, and the quenching effect of Cd on the fluorescence of two photosystems was detected to reflect the binding characteristic. The isolated PSI and PSII were resuspended in 0.05 M of phosphate buffer (pH = 7.4), and the chlorophyll concentration was adjusted to 10 μg Chl/mL. Three-dimensional fluorescence spectroscopy was obtained by setting the excitation and emission wavelengths from 350 to 550 nm and 450 to 850 nm, respectively. The wavelength ranges were set with a step of 5 nm, and the slit width of excitation and emission was 5 nm. After the test of fluorescence spectroscopy, the characteristic peak of two photosystems were determined. The excitation peak was at 436 nm, which was consistent with the fluorescence of chlorophyll material at room temperature. PSI and PSII have emission peaks at the same position around 685 nm. Thus, the fluorescence peak at EX436/EM685 was used to detect the quenching of the fluorescence of photosystems with Cd ions.

The resuspended solution of photosystems in 0.05 M phosphate buffer was used to test the quenching of the fluorescence, which was conducted in the cuvette set in the instrument. The concentration of Cd increased by 50 μM for each titration. During the titration, the solution was stirred for 15 min for equilibrium after the addition of 50 μM of heavy metal ions. Then, the fluorescence at EX436/EM685 was recorded after each titration. The excitation wavelength was set at 436 nm. The quenching parameters and binding constants were calculated by fluorescence intensity at the 685 nm. The measurement was repeated four times.

The equilibrium characteristics can be quantitatively described by the association constant (Ka) and binding site (n). These values of the fitting parameters could be obtained from the Lineweaver-Burk equation, as described by [48].

### 2.6. Statistics

Means and standard errors (S.E.) were calculated for each treatment in quadruplicate. The significance of differences between different treatments was performed by Analysis of Variance (one-way ANOVA). Significant difference was determined by Duncan’s test (*p* < 0.05).

## 3. Results

### 3.1. Effects of Cd on Growth

**Figure 1:**
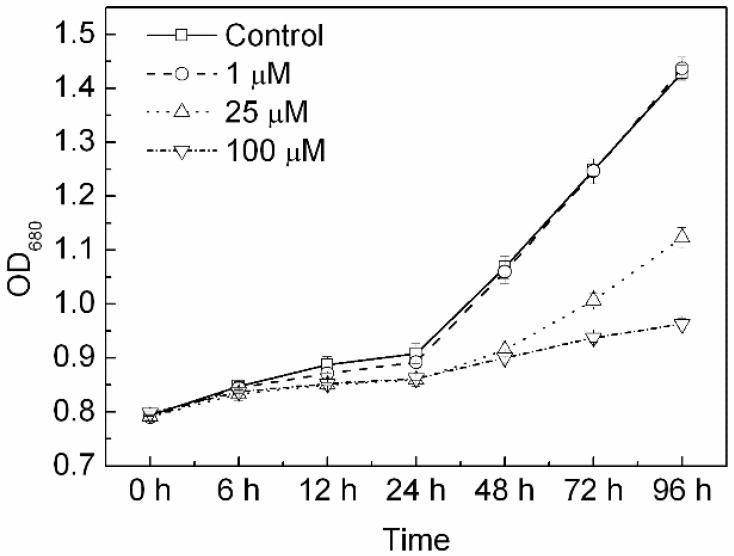
Cell growth of *Chlorella pyrenoidosa* at various Cd concentrations, expressed as the optical density at 680 nm (OD_680_). Data were recorded after the onset of treatments for 0, 6, 12, 24, 48, 72, and 96 h. All the data presented here were means and standard errors (S.E.) of quadruplicated samples.

The OD_680_ of all the treatments in the experiment kept increasing during the whole experiment (Figure 1). The OD_680_ of the cells treated with 1 µM Cd did not show a significant difference from control. After exposure to various concentrations of Cd for longer than 48 h, the cell growth rate decreased with the increasing Cd concentration. The cell growth rate of cells treated with 25 and 100 µM Cd was significantly lower than the control (*p* < 0.05, ANOVA, Duncan’s test).

### 3.2. Effects of Cd on Quantum Yields of Two Photosystems and CEF

**Figure 2:**
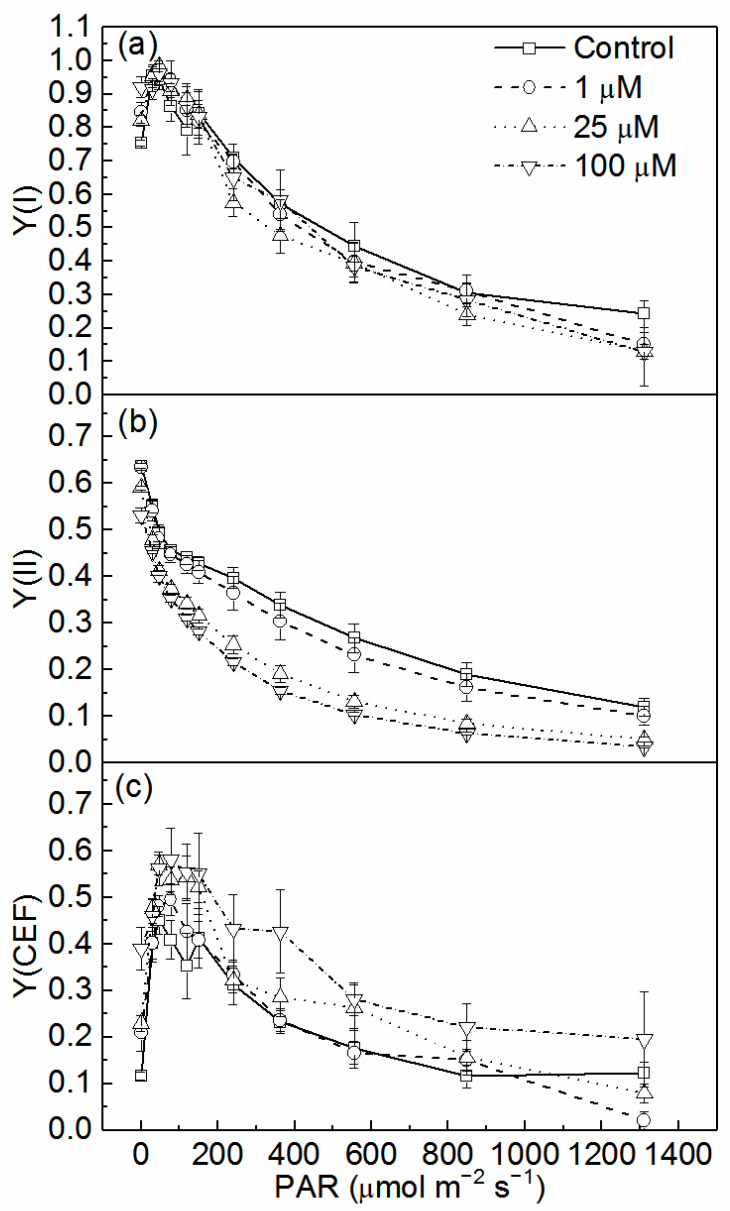
(**a**) The quantum yield of PSI [Y(I)], (**b**) the quantum yield of PSII [Y(II)], and (**c**) the quantum yield of CEF [Y(CEF)] of cells of *C. pyrenoidosa* exposed to various concentrations of Cd for 72 h. Data were detected during the rapid light response reaction. All the data presented here were means and standard errors (S.E.) of quadruplicated samples.

RLCs of Y(I), Y(II), and Y(CEF) were measured in the light response reaction after exposure to various concentrations of Cd for 72 h (Figure 2). Y(I) slightly increased at light intensity lower than 77 μmol m^−2^ s^−1^, then decreased with the increasing light intensity. Y(II) generally decreased with increasing PAR from 0, 30, 46, 77, 119, 150, 240, 363, 555, 849, to 1311 μmol m^−2^ s^−1^ (Figure 2a,b). The increase of Y(I) may be due to the increase of Y(CEF) at low light intensity (Figure 2c). Y(I) did not show a significant difference between different Cd treatments and the control during the entire light response reaction. Treatments with 25 and 100 μM Cd led to a significant decrease of Y(II) all the RLC (*p* < 0.05, ANOVA, Duncan’s test). Y(CEF) was significantly higher at 100 μM Cd than the control (*p* < 0.05, ANOVA, Duncan’s test).

### 3.3. Changes of Y(CEF)/Y(I), Y(II)/Y(I), and Y(CEF)/Y(II)

**Figure 3:**
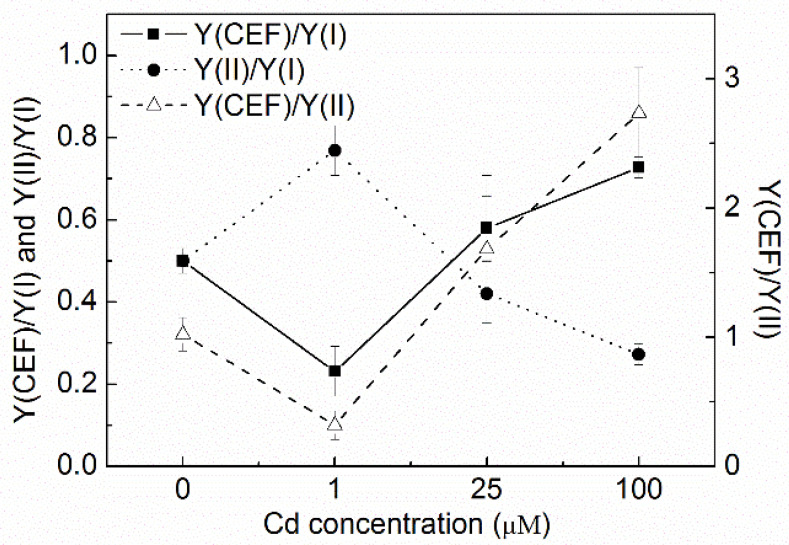
The changes of the ratios of Y(CEF)/Y(I), Y(II)/Y(I), and Y(CEF)/Y(II) of *C. pyrenoidosa* exposed to various concentrations of Cd for 72 h. Y(CEF)/Y(I) and Y(II)/Y(I) indicated the contribution of CEF and LEF to the yield of PSI. Y(CEF)/Y(II) indicated the ratio of quantum yield of CEF to LEF. Data were recorded after the last procedure of illumination at the highest intensity (1311 μmol m^−2^ s^−1^) during the rapid light response reaction. Data were presented as means and standard errors (S.E.) of quadruplicated samples.

Y(CEF)/Y(I) and Y(CEF)/Y(II) decreased after 1 µM of Cd treatment but largely increased with increasing Cd concentration. Y(CEF)/Y(I) and Y(CEF)/Y(II) of the cells treated with 100 µM of Cd were significantly higher than that of the control (*p* < 0.05, ANOVA, Duncan’s test). Y(CEF)/Y(II) increased from 1 for the control to around 3 for the 100 µM Cd treatment. In contrast, Y(II)/Y(I) generally decreased with the increasing Cd concentration. It decreased from 0.5 for the control to less than 0.3 for the cells treated with 100 µM Cd (Figure 3). After the cells were exposed to 100 µM Cd for 72 h, Y(CEF) contributed to most of Y(I), with an increase of Y(CEF)/Y(I) to above 0.7 and a decrease of Y(II)/Y(I) to less than 0.3.

### 3.4. Effects of Cd on Quantum Yields of Energy Conversion in PSI and PSII

**Figure 4:**
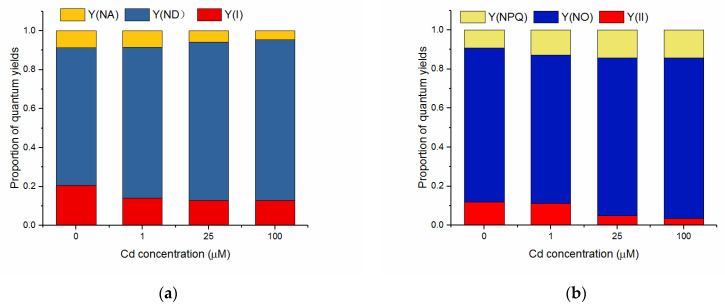
The complementary quantum yields of energy conversion in PSI (**a**) and PSII (**b**) of *C. pyrenoidosa* exposed to various concentrations of Cd for 72 h. Data were detected after the last procedure of illumination at the highest intensity (1311 μmol m^−2^ s^−1^) during the light response reaction. All the data presented were means of the quadruplicated samples.

The complementary quantum yields of energy conversion in PSI and PSII of algal cells untreated and treated with Cd for 72 h were shown in Figure 4. After the last procedure of illumination at the highest intensity (1311 μmol m^−2^ s^−1^) during the light response reaction, Y(I) and Y(ND) did not show a significant difference between the different treatments. Y(NA) decreased with the increasing concentration of Cd and was significantly lower when treated by 25 and 100 μM of Cd compared to the control (*p* < 0.05, ANOVA, Duncan’s test).

The photochemical quantum yield of PSII showed a concentration-dependent response to Cd, as indicated by the decrease of Y(II) with the increasing concentration of Cd. Y(II) of the cells treated with 25 and 100 μM of Cd were far lower than that of the control (*p* < 0.05, ANOVA, Duncan’s test). Y(NO) did not show a significant difference when the cells were treated with 1 and 25 μM of Cd but significantly increased at 100 μM of Cd compared to the control (*p* < 0.05, ANOVA, Duncan’s test). Y(NPQ) increased with increasing Cd concentration and was much higher than the control when the cells were treated with 25 and 100 μM of Cd (*p* < 0.05, ANOVA, Duncan’s test).

### 3.5. Effects of Cd on Electron Transport Rates in PSI and PSII

**Figure 5:**
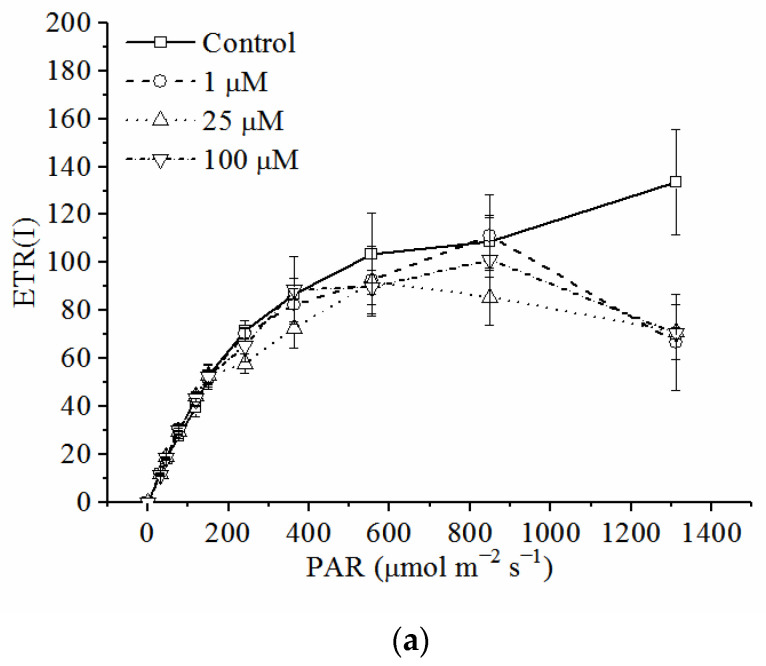

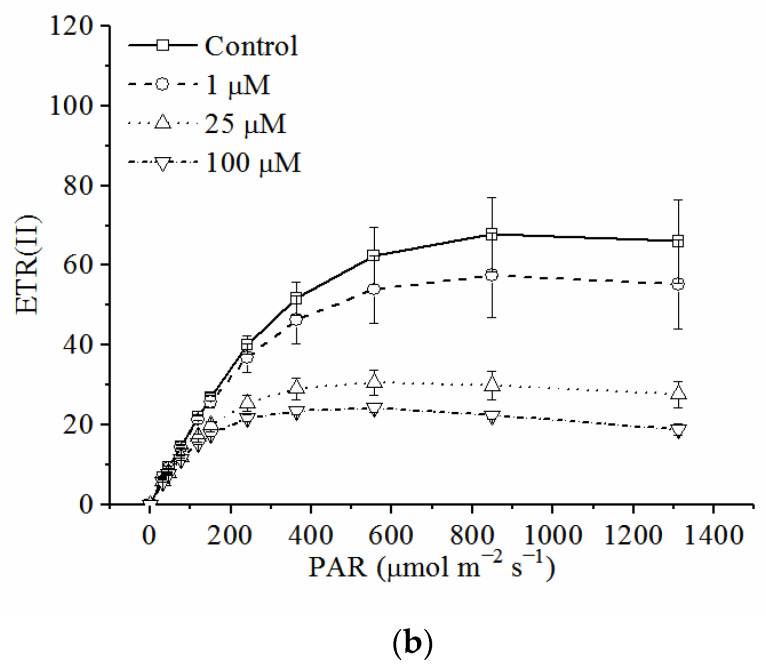
(**a**) The RLCs of ETR(I) and (**b**) the RLCs of ETR(II). Data were detected through the light response reaction after the cells of *C. pyrenoidosa* were exposed to various concentrations of Cd for 72 h. All the data presented were means and standard errors (S.E.) of the quadruplicated samples.

The RLCs of ETR(I) (Figure 5a) and ETR(II) (Figure 5b) after exposure to various concentrations of Cd for 72 h are shown in Figure 5. The RLCs of ETR(I) did not a show significant difference between the treatments and the control, whereas the RLCs of ETR(II) decreased with the increasing Cd concentration and were significantly lower for the treatments with 25 and 100 μM of Cd compared to the control (*p* < 0.05, ANOVA, Duncan’s test).

More information about the RLCs of electron transport rates in PSI and PSII could be derived from the descriptive parameters (Table 1). Ik, α, and ETRmax of the RLCs of ETR(I) did not show a significant difference between the different treatments and the control, whereas the parameters of the RLCs of ETR(II) showed obvious change due to the Cd treatments. Ik, α, and ETRmax decreased with the increasing Cd concentration and were significantly lower when treated by 25 and 100 μM of Cd compared to the control (*p* < 0.05, ANOVA, Duncan’s test). Ik, α, and ETRmax of the RLCs of ETR were higher in PSI than those of PSII in different treatments, except for Ik in the treatments with 0 and 1 μM of Cd.

### 3.6. Binding Ability of Cd Ions with Photosystems

**Figure 6:**
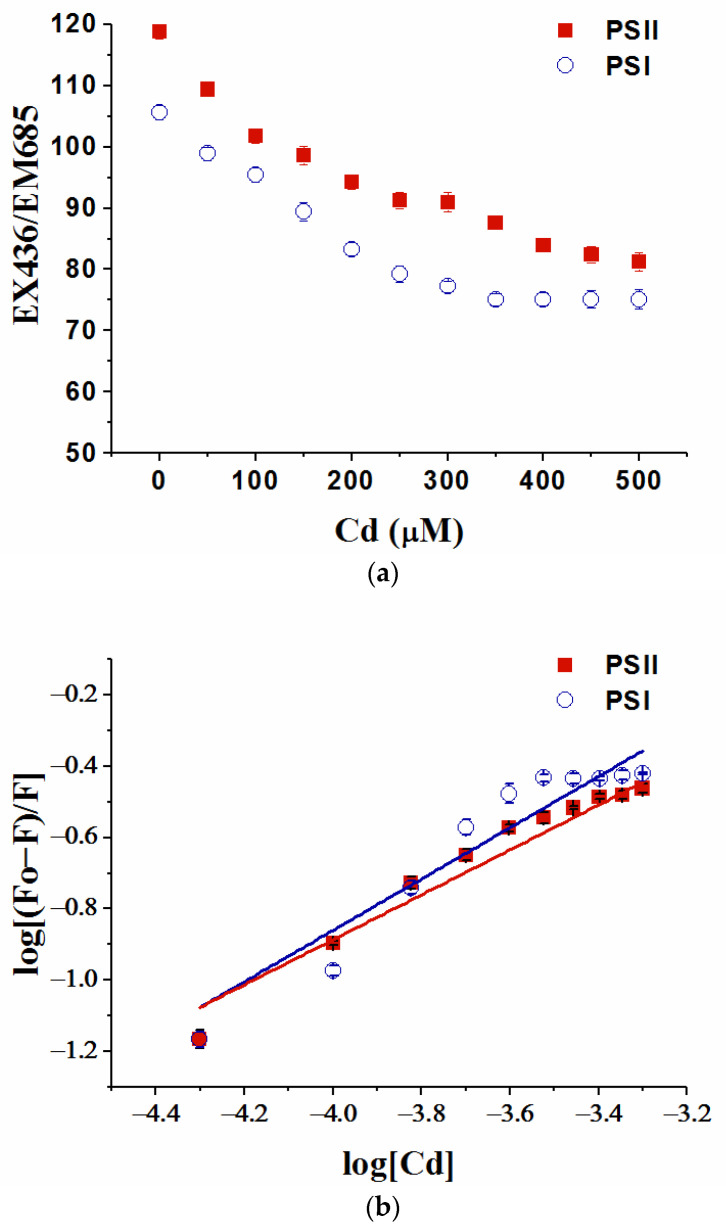
The binding process of Cd and photosystem particles isolated from *C. pyrenoidosa* detected by the quenching of the fluorescence. (**a**) The fluorescence intensity of photosystems at EX436/EM685 and the quenching of the fluorescence with Cd ions. (**b**) The equilibrium characteristics of the binding process by fitting of the fluorescence curves. Data were means of four replicates with standard errors.

The fluorescence intensity of photosystems and the quenching of the fluorescence with Cd ions are shown in Figure 6. Through the fluorescence quenching of the two photosystem particles with Cd, both the intensities of the fluorescence of the two photosystem particles posed significant quenching processes.

The fitting parameters of the quenching effects of heavy metals on the fluorescence are shown in Table 2. The association constant (Ka) showed that Cd has a strong binding ability with the two photosystems. There were more binding sites of Cd on PSII than that of PSI.

## 4. Discussion

In the present study, the toxic effects of Cd on cell growth and the activities of PSI, PSII, and CEF of *C. pyrenoidosa* were examined. Cell growth was reduced after exposure to Cd. The quantum yields of two photosystems and CEF were measured and defined in the Dual-PAM system as Y(I), Y(II), and Y(CEF), as described above. The electron transport rates in the two photosystems were also studied. Most previous studies investigated PSI and PSII separately. The photosynthetic activities of the two photosystems were analyzed simultaneously in the present study. Moreover, the binding ability of Cd and the photosystems was studied.

It has been confirmed by many studies that the cyclic flow of electrons around PSI is important for the photosynthesis of higher plants [22,33]. The cyclic electron flow around PSI is stimulated by many adverse environmental conditions, including drought and salt stress [19,49,50]. The existence and the characteristics of CEF in microalgae, which may live under different environmental stresses, were not well discussed previously. In the present study, the role of CEF in microalgae under environment stress was demonstrated.

Cd can cause inhibition of the activities of many enzymes and the growth, photosynthesis, or respiration in plant cells and algae [51,52]. Cd has been suggested to interact negatively with ATP production [53] and to decrease RNA and DNA synthesis, photosynthetic pigments, and proteins [54]. It was also reported that the decrease of metabolite turn over due to low photosynthetic productivity reduces cellular metabolites, including tricarboxylic acids, during Cd stress [55]. These effects lead to growth inhibition and even death due to Cd exposure. The inhibition of Cd of the growth of algae was also shown in the present study, indicated by the significant decrease of OD_680_ of the cells treated by 25 and 100 µM Cd for more than 48 h (Figure 1). The inhibitory effects of Cd on cell growth were associated with the inhibition of activity of photosynthesis, such as photochemical quantum yield and electron transport rate, as shown in the present study. It was worth noting that treatment with Cd at low concentrations (1 µM) showed no inhibition of the cell growth rates of *C. pyrenoidosa*. This may be due to a variety of detoxification mechanisms in algae, even if they absorb and accumulate Cd, and algae could take advantage of their ability to cope with high intracellular amounts of heavy metals to overcome the stress [56].

The photosynthetic membranes are very sensitive to Cd, and Cd was suggested to firstly affect chlorophyll content, then inhibit the photochemical activity of PSII and oxygen-evolving complex (OEC), and later the PSI activity [24]. Both PSII and PSI experienced photoinhibition under Cd stress, which was in accordance with some previous studies [57,58]. From the rapid light response reaction of Y(I) and Y(II) of the cells with different treatments (Figure 2), the photochemical quantum yield of PSII were more susceptible to Cd treatment, which agreed with PSII being a sensitive site to many environmental stresses [15,59,60], and PSI photochemistry was more stable than PSII photochemistry as in some studies [30,31]. The results of this study are consistent with the data of barley under cadmium stress, which showed that Cd treatment increased the donor-side limitation, i.e., Y(ND), and decreased Y(I) [58]. Compared to the widely used chlorophyll fluorescence parameters Fv/Fm (maximum quantum yield of PSII), the higher sensitivity of Y(II) to Cd stress was suggested by previous studies [61,62]. The sensitivity of Y(II) and its significant reduction under Cd stress was also exhibited in the present study. Y(NPQ) increased with the decrease of Y(II), which suggested that, under Cd stress, more absorbed energy flux was dissipated via regulated non-photochemical quenching in PSII to avoid over-reduction of the electron transport chain. These processes represent an important role that was similar to that of the photoprotective mechanism in plants [63].

Y(I) comprises Y(II) and Y(CEF) as the calculation referring to Huang et al. (2010) [31]. Both Y(I) and Y(II) decreased with the increase of the light intensity due to photoinhibition. Y(I) showed a slight increase under low light (lower than 77 μmol m^−2^ s^−1^) (Figure 2a), which may mainly be due to the increase of Y(CEF) (Figure 2c). Some research suggested that PSI photochemical activity in excess light is more stable than PSII photochemistry [30,64], and PSI turnover saturates at a higher irradiance than PSII, due to the occurrence of CEF [22]. In the present study, the contribution of CEF to the stability of PSI has also been proposed, which is indicated by the lesser decrease of Y(I) after Cd treatments (Figure 2). This was in agreement with the results that the activation of CEF when treated by Cd could reduce the acceptor side limitation of PSI, as indicated by the decrease of Y(NA) (Figure 4), and protect PSI [32,34].

The change of quantum yield distribution between the photosystems and the relation of CEF and LEF could be derived from the change of Y(CEF)/Y(I), Y(II)/Y(I), and Y(CEF)/Y(II) (Figure 3). The significant decrease of Y(II) caused the decrease of the ratio of Y(II)/Y(I), suggesting an imbalanced distribution of quantum yields between the photosystems. This confirmed that Cd inhibited PSII more than PSI, together with the results from the quantum yields of energy conversion of two photosystems (Figure 4). Y(II) decreased significantly and Y(CEF)/Y(II) increased significantly in the cells treated with 100 µM Cd, indicating the activation of CEF and the inhibition of LEF. The activation of CEF was also indicated by the increase of Y(CEF)/Y(I). The results showed that Y(CEF) made a big contribution to Y(I), especially when the cells were treated with a high concentration of Cd (100 µM). Zhou et al. (2006) [8] detected an increase of PSI activity in *M. aeruginosa* treated with elevated levels of cadmium, which is probably linked to the enhancement of CEF around PSI. The results in the present study confirmed that the existence and activation of CEF could increase the tolerance of PSI in *C. pyrenoidosa* to Cd and keep the stability of PSI.

As discussed above, CEF was activated when the cells were treated with Cd. NPQ was also enhanced due to Cd treatment, which could be indicated by the quantum yield of light-induced non-photochemical fluorescence quenching, Y(NPQ) (Figure 4). CEF was suggested to enhance the generation of a pH gradient across the thylakoid membrane (ΔpH), which was important for the synthesis of ATP and the induction of NPQ [30]. The enhancement of ATP generation may provide more energy in the protection of the photosynthetic apparatus of *C. pyrenoidosa*, such as energy for the production of metal-binding polypeptides [65] and the synthesis of enzymes and compounds related to oxidative stress [66].

Our previous research preliminarily revealed the effects of Cd on the quantum yields of two photosystems [35], but more information on the effects could be derived from the RLC of ETR in the present study. The electron transport rate in PSII was more susceptible to Cd (Figure 5). The amplitude of ETR(II) decreased significantly when treated with 25 and 100 μM Cd. Fitting parameters of RLCs showed more details about the photoinhibitory effects of Cd on the electron transport of PSII and PSI. Cd reduced the efficiency of PSII to use the energy under high light, which was indicated by the decrease of Ik, α, and ETRmax with the increasing Cd concentration (Table 1). However, the activation of CEF was useful for the stability of ETR in PSI when the cells were treated with Cd, indicated by the little difference in Ik, α, and ETRmax of ETR(I) between different treatments (Figure 5, Table 1). These results demonstrated the importance of CEF under Cd stress. This work also showed the advantage of RLC of ETR in the toxicological analysis [62].

It was suggested that metal toxicity on photosynthesis is related to the binding ability of the photosynthetic apparatus to metals [67]. Therefore, the binding reaction between photosystems and heavy metal ions may explain their different toxicity. Through the application of fluorescence spectroscopy, we can analyze the migration characteristics of environmental pollutants in different environmental media and the complexation process of bioorganic substances and heavy metal ions [68,69]. It is reasonable to think that there are different binding sites between photosystems and Cd. In this experiment, the binding process of Cd ion and photosystems was preliminarily studied by the quenching of fluorescence. However, the specific binding position was neither clear nor distinguished in the present study. Although the results may not be entirely accurate, the photosystems were considered as a whole to conduct the binding experiment and the Lineweaver-Burk equation, which was used for fitting and analysis. The meaning of the fitting and the results was just for the comparison of the binding strength and the relative quantity of binding sites in the present study. Through the analysis of the quenching effects of Cd on the fluorescence of the two photosystems from *C. pyrenoidosa*, the strong binding ability of Cd and the two photosystems was found. This result explained the high toxicity of Cd to the growth of alga and photosystem apparatus, while the binding sites of PSII and Cd were more than PSI. This was consistent with the results that Cd significantly inhibited the quantum yield and electron transfer activity of PSII rather than those of PSI.

## 5. Conclusions

In the present study, exposure to Cd inhibited both cell growth and activities of the photosynthetic apparatus of *C. pyrenoidosa*. The inhibition of Cd of cell growth was associated with the inhibition of photosynthesis. In comparison with PSI, PSII was more susceptible to Cd treatment. The effective photochemical quantum yield and electron transport rate in PSII were seriously inhibited by Cd, whereas CEF was activated due to Cd treatment and contributed to the photochemical quantum yield and stability of electron transport rate in PSI. The present study confirmed that CEF functioned in the tolerance of PSI to Cd treatment. The activation of CEF also played an important role in the induction of non-photochemical quenching (NPQ). The activation of CEF and the induction of NPQ were important protective mechanisms used by *C. pyrenoidosa* to overcome Cd toxicity. As in some studies, the Dual-PAM-100 system, which could measure chlorophyll fluorescence and P700^+^ absorbance changes simultaneously, was useful for studying the energy transport in the photosynthetic apparatus and the toxic effects of pollutants on photosynthetic organisms. The binding characteristics of Cd ions to photosystem particles may explain the different toxicity of Cd to PSII and PSI.

## Figures and Tables

**Table 1:** Descriptive parameters of the light response reaction were derived from the rapid light curves (RLCs) of ETR(I) and ETR(II).

Concentration of CdCl_2_ (μM)	Parameters of the Light Curve of ETR(I)			Parameters of the Light Curve of ETR(II)		
	Ik (μmol photon m^−2^ s^−1^)	α (e^−^ photon^−1^)	ETRmax (μmol e^−^ m^−2^ s^−1^)	Ik (μmol photon m^−2^ s^−1^)	α (e^−^ photon^−1^)	ETRmax (μmol e^−^ m^−2^ s^−1^)
0	257.3 ± 38.1 ^a^	0.423 ± 0.050 ^a^	133.0 ± 21.5 ^a^	304.8 ± 40.3 ^a^	0.226 ± 0.005 ^a^	69.3 ± 9.9 ^a^
1	240.0 ± 35.9 ^a^	0.434 ± 0.015 ^a^	102.9 ± 13.1 ^a^	256.6 ± 43.9 ^a^	0.226 ± 0.005 ^a^	58.4 ± 11.0 ^a^
25	209.4 ± 23.2 ^a^	0.439 ± 0.035 ^a^	89.8 ± 6.1 ^a^	150.2 ± 16.0 ^b^	0.205 ± 0.002 ^b^	30.9 ± 3.4 ^b^
100	202.4 ± 8.8 ^a^	0.394 ± 0.054 ^a^	89.1 ± 7.4 ^a^	123.9 ± 4.0 ^b^	0.196 ± 0.003 ^b^	24.3 ± 1.0 ^b^

Note: The data were automatically calculated by the Dual-PAM software. The measurements were carried out after the cells of *C. pyrenoidosa* were exposed to various concentrations of Cd for 72 h. All the data presented were means and standard errors (S.E.) of quadruplicated samples. Data followed by different letters in the same column are significantly different (*p* < 0.05, ANOVA, Duncan’s test).

**Table 2:** Fitting parameters of quenching curves of the fluorescence of photosystem particles isolated from *C. pyrenoidosa* by the titration of Cd.

Heavy Metal	PSI Particles		PSII Particles	
	Ka (×10^4^ M^−1^)	n	Ka (×10^4^ M^−1^)	n
Cd	2.01 ± 0.18	0.52 ± 0.09	1.95 ± 0.06	0.77 ± 0.13

Note: Data were derived from four measurements and shown as means and standard errors (S.E.).

## Data Availability

The datasets used or analyzed during the current study are available from the corresponding author on reasonable request.
